# Urinary Microbiota Associated with Preterm Birth: Results from the Conditions Affecting Neurocognitive Development and Learning in Early Childhood (CANDLE) Study

**DOI:** 10.1371/journal.pone.0162302

**Published:** 2016-09-09

**Authors:** Nicholas J. Ollberding, Eszter Völgyi, Maurizio Macaluso, Ranjit Kumar, Casey Morrow, Frances A. Tylavsky, Chandrika J. Piyathilake

**Affiliations:** 1 Division of Biostatistics and Epidemiology, Cincinnati Children’s Hospital Medical Center, Cincinnati, Ohio, United States of America; 2 Department of Pediatrics, The University of Tennessee Health Science Center, Memphis, Tennessee, United States of America; 3 Department of Preventive Medicine, The University of Tennessee Health Science Center, Memphis, Tennessee, United States of America; 4 University of Alabama at Birmingham, Center for Clinical & Translational Science, Birmingham, Alabama, United States of America; 5 University of Alabama at Birmingham, Department of Cell Developmental and Integrative Biology, Birmingham, Alabama, United States of America; 6 University of Alabama at Birmingham, Department of Nutrition Sciences, Birmingham, Alabama, United States of America; Medical University of South Carolina, UNITED STATES

## Abstract

Preterm birth (PTB) is the leading cause of infant morbidity and mortality. Genitourinary infection is implicated in the initiation of spontaneous PTB; however, examination of the urinary microbiota in relation to preterm delivery using next-generation sequencing technologies is lacking. In a case-control study nested within the Conditions Affecting Neurocognitive Development and Learning in Early Childhood (CANDLE) study, we examined associations between the urinary microbiota and PTB. A total of 49 cases (delivery < 37 weeks gestation) and 48 controls (delivery ≥ 37 weeks gestation) balanced on health insurance type were included in the present analysis. Illumina sequencing of the 16S rRNA gene V4 region was performed on urine samples collected during the second trimester. We observed no difference in taxa richness, evenness, or community composition between cases and controls or for gestational age modeled as a continuous variable. Operational taxonomic units (OTUs) classified to *Prevotella*, *Sutterella*, *L*. *iners*, *Blautia*, *Kocuria*, Lachnospiraceae, and *S*.*marcescens* were enriched among cases (FDR corrected *p*≤ 0.05). A urinary microbiota clustering partition dominated by *S*. *marcescens* was also associated with PTB (OR = 3.97, 95% CI: 1.19–13.24). These data suggest a limited role for the urinary microbiota in PTB when measured during the second trimester by 16S rRNA gene sequencing. The enrichment among cases in several organisms previously reported to be associated with genitourinary pathology requires confirmation in future studies to rule out the potential for false positive findings.

## Introduction

Preterm birth (PTB) is the leading cause of infant morbidity and mortality [1], [2] and occurs in approximately 11% of all live births.[2–4] Microbial infection of the genitourinary and reproductive tract during pregnancy is thought to be an initiating factor in spontaneous PTB. Intrauterine infections, bacterial vaginosis (BV), urinary tract infections (UTI), and maternal systemic infections have been associated with preterm delivery.[5–8] A prevailing theory postulates that pathogenic organisms of the lower genital tract migrate to the fetal membranes, and subsequently into the amniotic fluid, invoking an inflammatory response that results in the initiation of preterm labor.[9–11] However, recent reports have also suggested hematogenus transmission of microbes to the amniotic fluid providing a plausible mechanism for systematic infection in PTB.[12–15]

Numerous organisms including *Ureaplasma* spp., *G*. *vaginalis*, and *Mycoplasma* spp. have been found in the amniotic tissue or fluid of preterm samples by culture or PCR.[16] Culture-dependent approaches have also identified BV-associated species including *Prevotella* spp., *G vaginalis*, and *Peptostreptococcus* spp. to be associated with preterm delivery.[16] However, these methods are limited to identifying only a fraction of the bacterial diversity of female genitourinary and reproductive tract. Moreover, the disappointing results of antibiotic treatment for the prevention of PTB [17] may, in part, be related to the lack of comprehensive assessment of pathogenic microbes associated with preterm delivery. The increased accessibility and reduced cost of next-generation sequencing (NGS) platforms now provides the opportunity to examine unculturable and rare organisms and to assess microbial community composition. Culture-independent investigations of the cervicovaginal microbiota have reported PTB to be associated with increased α-diversity, as well as a high diversity, *Lactobacillus* poor community state type; although, results have been inconsistent.[11, 18–21]

Recent efforts using culture-independent approaches have shown high diversity and number of operational taxonomic units (OTUs), as well as the presence of opportunistic, anaerobic bacteria associated with female urogenital pathology in the urine of asymptomatic women.[22–27] In particular, *Lactobacillus*, *Prevotella* and *Gardnerella* organisms have been shown to be dominant genera of the female urinary microbiota.[23] Thus, the use of NGS technologies may provide for novel insight into the role of the female urinary microbiota in the etiology of PTB by allowing for the examination of large numbers of previously unobservable organisms.

For the present nested case-control study, we examined whether bacterial diversity and community composition identified from 16S rRNA gene sequencing of urine collected during the second trimester were associated with the risk of PTB among women participating in the Conditions Affecting Neurocognitive Development and Learning in Early Childhood (CANDLE) study. We also examined whether specific OTUs generated from percent similarity and entropy-based partitioning methods or the inferred metagenome were associated with delivery status.

## Materials and Methods

### Study Population

The Urban Child Institute's Conditions Affecting Neurocognitive Development and Learning in Early childhood (CANDLE) project was designed to provide insights into the biological and environmental factors that influence development during the first years of life. The CANDLE project consists of a racially and socioeconomically diverse cohort of approximately 1,500 mother-child dyads.[28] Briefly, between December 2006 and July 2011, women in the second trimester of a singleton pregnancy; residing in Memphis and Shelby County, TN; age 16–40 years at enrollment; and intending to deliver at a participating hospital were eligible for participation. Exclusion criteria were existing chronic disease requiring medical treatment or pregnancy complications including premature rupture or prolapse of membranes, placenta previa, or oligohydraminios prior to enrollment. A total of 135 mothers (9%) experienced uninduced preterm deliveries (<37 weeks). A random subset (*n* = 50) were selected for the present analysis. An additional 50 mothers enrolled in the CANDLE study who delivered at term (>37 weeks) and were balanced on health insurance type as a proxy for socioeconomic status were randomly selected as controls. Written informed consent was obtained from participants 18 years of age. Assent and parental or guardian written consent were obtained from all participants less than 18 years of age. The study protocol and informed consent process was approved by the institutional review board of the University of Tennessee Health Sciences Center.

### Data and Sample Collection

Demographic, health, and pre-pregnancy anthropometric information was collected via self-reported questionnaires at enrollment. Nurse coordinators abstracted information concerning labor and delivery from the medical records. Maternal urine (60mL) specimens were collected into 120cc plastic containers, aliquoted, and kept cold (2–4°C) and frozen (-70°C) within 1 hour of collection. Participants were provided with Benzalkonium (BZK) wipes and instructed to wipe from front to back and then collect the urine. 2mL aliquots were mailed on dry ice to the laboratory of Piyathilake and kept frozen at -70°C until microbial DNA was isolated.

### DNA Extraction, Amplicon Library Preparation, and Sequencing

Microbial DNA was isolated using the Fecal DNA isolation kit from Zymo Research (Irvine, CA). The V4 region of the 16S rRNA gene (515F/806R) was amplified from extracted DNA using the protocol and region-specific PCR primers described by Caparoso et al.[29] PCR products were purified by gel electrophoresis and quantitated using the Picogreen (Invitrogen) dsDNA quantitation assay prior to sequencing. Paired end sequencing (2 x 250) was performed on the Illumina MiSeq platform (Illumina Inc., San Diego, CA).

### Data Processing

Sequence reads were quality filtered and processed using an integrated, high-throughput 16S rDNA analysis pipeline built on the QIIME v1.8 tool suite.[30] Read filtering and processing included: initial quality check of raw sequencing data (FASTQC) [31], quality filtering (FASTX-Toolkit) [32], merging of paired-end reads (QIIME), chimera filtering (UCHIME) [33], cluster de novo OTUs at 97% similarity (UCLUST) [34], assign taxonomy (RDP Classifier) [35] using the Greengenes v13.8 reference database [36], align sequences (PyNAST) [37] and construct a phylogenetic tree (FastTree).[38] Filtered OTU tables were obtained by removing OTUs with an abundance < 0.005% [39] and subsampling to 20k reads; resulting in the exclusion of *n* = 3 participants due to low read counts. Phylogenetic Investigation of Communities by Reconstruction of Unobserved States (PICRUSt) [40] was used to infer metagenome functional gene content from reference-assisted, 16S-derived community composition. Measures of alpha-diversity (observed richness, Shannon Index [41] and Faith’s phylogenetic diversity [42]) were obtained using QIIME. Beta-diversity metrics (UniFrac [43], Bray-Curtis [44], Jaccard [42], Morisita-Horn [45]) were calculated as implemented in the R package vegan.[46] Minimum Entropy Decomposition (MED) was also used to partition sequence reads into MED nodes.[47] MED uses Shannon entropy to iteratively partition sequence reads based on information-rich (high entropy) positions while accounting for stochastic variation via abundance filtering. Resolution to a single nucleotide is possible with MED and was chosen to allow for discrimination between closely related organisms while limiting artificial variation. MED was applied to the merged reads after filtering those < 200bp in length and minimum quality score < 25. MED partitioning was performed using the default settings with the–M noise filter set to 385 (7,697,666/20k) resulting in 17% of reads filtered and 810 MED nodes. Taxonomic assignment of MED nodes was performed by manual BLAST against the NCBI reference database.

### Statistical Analysis

The first objective was to compare cases and controls on α- and β-diversity. Differences in demographic and clinical characteristics were assessed by t-tests or Wilcoxon rank-sum tests for continuous variables and χ2 tests for categorical variables. We compared α-diversity metrics using the Wilcoxon rank-sum test with differences in β-diversity examined by non-metric dimensional scaling (NMDS) and tested by permutational ANOVA.[46] Differences in the PICRUSt imputed metagenome were also examined by NMDS and tested by permutational ANOVA. Dirichlet multinomial models were used to test for differences in phyla- and genus-level phylotypes with expected frequencies under the Dirichlet multinomial model obtained using the HMP package in R and tested by the χ2 test of location.[48]

The second objective was to evaluate associations for OTUs and MED nodes with PTB. We used negative-binomial regression as implemented in the DESeq2 [49] and package in R and phyloseq interface [50] to test for differences between cases and controls. Models were fit to the data without subsampling. Multivariable models and included adjustment for covariates as described below. Log2-fold changes for OTUs and MED nodes with false discovery rate (FDR) corrected p ≤ 0.05 are reported.

Our third objective was to assess whether we could identify naturally occurring clusters of participants based on the co-occurrence of abundant organisms in the urinary microbiota and assess their association with PTB. A Dirichlet multinomial mixture (DMM) model was used to cluster samples into partitions.[51] The DMM model as implemented in Mothur v1.35 [52] was fit to the most abundant (n = 20) genus-level phylotypes to improve clustering. Optimal partition number was chosen based on minimizing the Laplace approximation to the negative log model evidence. The DMM provides an evidence-based approach to the estimation of metacommunities, as well as parameters for the weight (i.e., relative frequency, π) and precision (i.e., a measure inversely related to the degree of dispersion with respect to OTU composition, θ) of each partition. Odds ratios (OR) and 95% confidence intervals (CI) for preterm delivery according to urinary microbiota clustering partitions were calculated using logistic regression. OR were calculated by contrasting each partition to samples collected from all other women. Multivariable models included adjustment for maternal age, body mass index, weight gain during pregnancy, race (black vs. non-black), income (<$20k/y, $20-$45k/y, >$5k/y), education (<high school, high school or technical college, college graduate), abnormal vaginal discharge at delivery (yes/no), Group B streptococcus infection at delivery (yes/no) and the history of PTB (yes/no). Multiple imputation (*n* = 30 datasets) was performed for missing covariate data using the Amelia II package [53] in R.

## Results

### Participant Characteristics

Mean maternal age at delivery was 25.7 (±5.2) years, 62.9% identified as non-Hispanic black, 39.2% were college graduates, and 35.1% reported a maternal income exceeding $45k/annually (Table 1). Mean parity was 2.5 (±1.4), pre-pregnancy body mass index was 27.1 (±7.6), and weight gain during pregnancy was 14.5 (±6.9) kg. Cases were older than controls (cases = 26.8 y, controls = 24.5 y; *p* = 0.03), but did not differ on other demographic or clinical characteristic examined. Notably, a history of PTB was more common among cases than among controls, but failed to reach statistical significance (*p* = 0.09). Infant gestational age at birth was 34.8 (±2.3) and 39.2 (±1.1) weeks for cases and controls, respectively (*p*<0.01).

**Table 1:** Characteristics of mothers and infants according to delivery status.

Characteristic	Preterm (*n* = 49)	Term (*n* = 48)	p-value*
Maternal age y, mean (SD)	26.8 (5.4)	24.5 (4.8)	0.03
Maternal race/ethnicity, n (%)†			
Non-Hispanic black	32 (65.3)	29 (60.4)	0.82
Non-Hispanic white	16 (32.7)	16 (33.3)	
Other	1 (2.0)	3 (6.3)	
Maternal education, n (%)			
< High school	7 (14.3)	7 (14.6)	0.62
High school diploma or technical degree	25 (51.0)	20 (41.7)	
College graduate	17 (34.7)	21 (43.8)	
Maternal income, n (%)			
< $20,000 y	17 (34.7)	10 (20.8)	0.46
$20,000–< $45,000 y	9 (18.4)	11 (22.9)	
≥ $45,000 y	18 (36.7)	16 (33.3)	
Marital status, n (%)			
Married	18 (36.7)	19 (39.6)	0.95
Unmarried (living alone)	22 (44.9)	21 (43.8)	
Unmarried (living with partner)	9 (18.4)	8 (16.7)	
Total number of pregnancies, mean (SD)	2.8 (1.6)	2.1 (1.1)	0.08
History of previous preterm delivery, n (%)	8 (16.3)	2 (4.2)	0.09
Pre-pregnancy BMI, mean (SD)	27.4 (8.2)	26.9 (6.9)	0.91
Total weight gain during pregnancy (kg), mean (SD)	15.3 (7.4)	13.4 (6.1)	0.41
Infant birth length (cm), mean (SD)	46.0 (4.4)	50.8 (2.3)	< .01
Infant birth weight (g), mean (SD)	2438.8 (656.1)	3372.4 (425.0)	< .01
Infant gestational age at birth (weeks), mean (SD)	34.8 (2.3)	39.2 (1.1)	< .01
Maternal abnormal vaginal discharge at birth, n (%)	9 (18.4)	6 (12.5)	0.42
Group B streptococcus infection at birth, n (%)	9 (18.4)	10 (20.8)	0.33

Abbreviations: SD, standard deviation; BMI, body mass index (kg/m2).

Note: Values may not sum to total due to missing data.

*P-value for t-test or Wilcoxon rank-sum test for continuous variables and chi-square test for categorical variables.

^†^Comparison for non-Hispanic black and non-Hispanic white only.

### Microbial Composition and PTB

**Fig 1:**
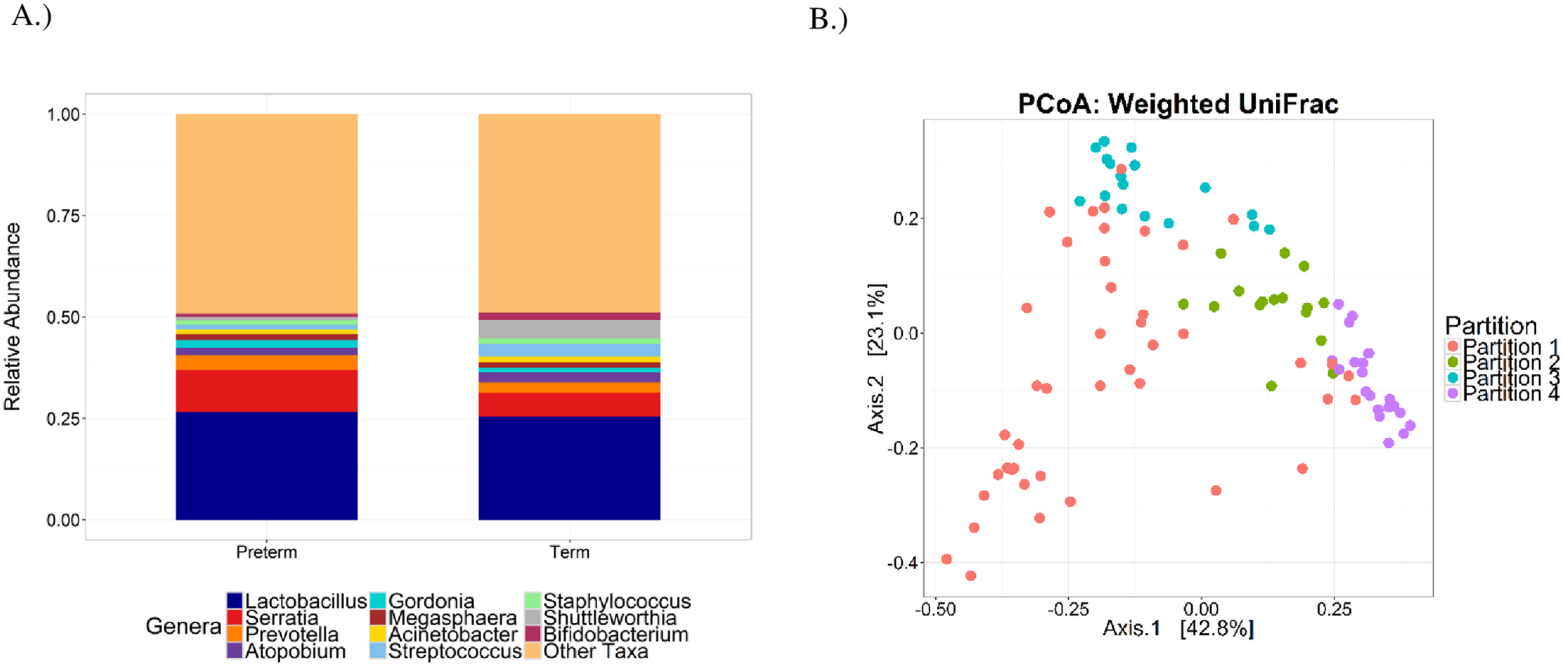
Urinary microbiota community composition. (A) Abundant genus-level phylotypes according to delivery status. (B) Principal coordinates ordination of the weighted UniFrac metric (variance axis 1 = 42.8%, variance axis 2 = 23.1%; *p*<0.001) for clustering by partitions identified from the Dirichlet multinomial model.

**Fig 2:**
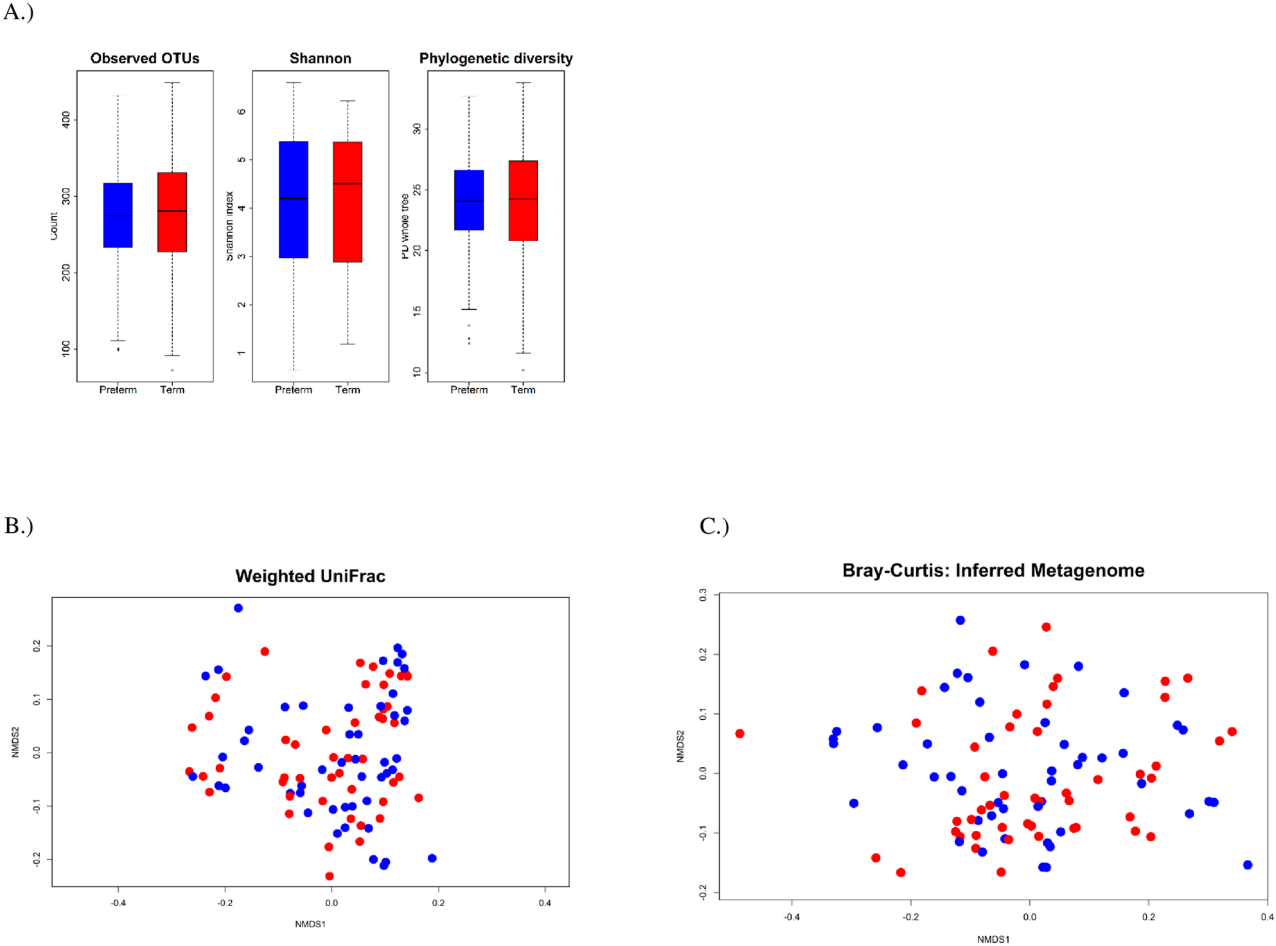
α-diveristy estimates and non-metric dimensional scaling (NMDS) comparing community compostion as meaured by the weighted UniFrac metric and Bray-Curtis dissimialrty for the inferred metagenome for mothers delivering at term (red) or preterm (blue). (A) Box-plots for α-diveristy estimates (*P* value >0.76 for all models). (B) NMDS of the weighted UniFrac metric (stress = 0.16; *p* = 0.78 for clustering by PTB). (C) NMDS of the Bray-Curtis dissimialrity on the inferred metagenome (stress = 0.11; *p* = 0.82 for clustering by PTB).

Genus-level phylotypes are shown in Fig 1 according to delivery status. *Lactobacillus*, *Serratia*, *Prevotella*, *Atopobium*, and *Gordonia* organisms were among the most abundant genera detected. The Dirichlet parameter test did not support differences in the most abundant genera between cases and controls (*p*>0.82). Similar results were obtained for phyla (*p*>0.78, data not shown). Large numbers of reads were also classified to Bifidobacteriacea (13.4%), Caulobacteraceae (3.7%), and Oxalobacteraceae (2.4%) with the relative abundance similar for cases and controls. Observed OTU richness was 275 (IQR = 84) for cases and 280 (IQR = 103) for controls (*p* = 0.99, Fig 2, S1 Table). Additional α-diversity metrics did not support differences in richness or evenness (*p*> 0.88); nor did ordinations and permutational ANOVA performed on dissimilarity matrices obtained from UCLUST OTUs, MED nodes, or the imputed metagenome (*p*>0.47, Fig 2, S2 Table). No associations were detected for α- or β-diversity metrics in sub-analyses restricted to early (< 34 weeks; *n* = 13) and late (34–36 weeks, *n* = 36) PTB or for gestational age when modeled as a continuous variable (data not shown).

### Associations for OTUs and MED Nodes

Differential abundance testing identified enrichment in both UCLUST OTUs and MED nodes mapping to *Prevotella*, *Sutterella/Parasutterella*, and Lachnospiraceae organisms in the urine of cases (FDR corrected *p*≤0.05, S3 and S4 Tables). Enrichment in *Streptococcus*, *S*. *agalactiae*, *A*. *vaginae*, and *Shuttleworthia* was observed in the urine of controls. Large inverse associations were seen for an abundant OTU/node classified to *Shuttleworthia*. There were also inconsistencies for partitioning approaches. MED identified *L*. *iners* and two nodes classified to *Blautia* (one *to B*. *obeum)* as enriched in cases. Interestingly, MED partitioning identified several small nodes classified to *Gardnerella* as enriched in cases, whereas this association was not seen for the UCLUST OTUs. Similarly, associations were observed for UCLUST OTUS, and not MED nodes, including a positive association for an OTU classified to *Kocuria*. A UCLUST OTU (log2fold = 0.76, SE = 0.26, FDR *p* = 0.05) and MED nodes (log2fold = 0.90, SE = 0.31, FDR *p* = 0.05) mapping to *S*.*marcescens* were also enriched in cases. A total of 34 MED nodes and 15 UCLUST OTUs were associated with delivery status after false discovery rate correction.

### Urinary Microbiota Clustering Partitions and PTB

Four partitions were identified from the DMM model (Table 2). Partition 1 was the most prevalent, had the lowest clustering precision, and was uniquely characterized by the high proportion of reads classified to Bifidobacteriacea (24%), *Prevotella* (6%), and *Shuttleworthia* (6%) (S5 Table). Partition 2 had the highest clustering precision and was dominated by *Lactobacillus* (29%) followed by Bifidobacteriacea (10%) organisms. Partition 3 was small with low precision and uniquely characterized by the high proportion of reads classified to *S*. *marcescens* (26%). Partition 4 was the least varied and dominated by *Lactobacillus* organisms (73%). Together the partitions explained 35% of the variance in the composition of the urinary microbiota as measured by the weighted UniFrac distance matrix (*p*<0.001). In unadjusted models, the odds ratio for PTB was nearly 4-fold higher (OR = 3.97 [1.19; 13.24]) for samples clustered to partition 3 when compared to all other samples. The odds ratio was increased when adjusted for factors contributing to preterm delivery; however, estimates for both models were based on a small number of cases and imprecise. No associations were detected for the other partitions. Mothers in partition 3 delivered 1.7±0.97 (p = 0.08) weeks earlier than those in partition 2. Sensitivity analyses were also conducted to examine associations for early (< 34 weeks; *n* = 13) and late (34–36 weeks, *n* = 36) PTB separately. The odds ratio for early PTB was OR = 3.30 (0.64; 17.13) and for late PTB the OR = 4.23 (1.20; 14.87) for samples clustered to partition 3 when compared to all other samples. Similar associations were obtained in models adjusted for factors contributing to preterm delivery.

**Table 2:** Odds ratios and 95% confidence intervals for preterm birth according to urinary microbiota community type.

Community type	Partition 1	Partition 2	Partition 3	Partition 4
Cases/Controls	19/24	6/10	13/4	11/10
π (θ)*	0.44 (7.4)	0.17 (54.2)	0.17 (34.7)	0.21 (74.1)
Unadjusted model	0.63 (0.28; 1.42)	0.53 (0.18; 1.60)	3.97 (1.19; 13.24)	1.10 (0.42; 2.89)
Multivariable model†	0.61 (0.20; 1.83)	0.38 (0.09; 1.63)	4.49 (1.01; 19.90)	1.19 (0.35; 4.03)

Notes: Partitioning of samples was performed using a Dirichlet multinomial mixture model. Odds ratios reported for contrast of partition to all other partitions (e.g. partition 1 vs. partitions 2–4).

Missing covariate data in adjusted models was multiply imputed (n = 30 datasets) using the Amelia II package in R.

*π reflects proportion of samples clustered to the partition. θ reflects precision with small values reflecting greater variation.

^†^Adjusted for maternal age, body mass index, weight gain during pregnancy, race (black vs. non-black), income (<$20k/y, $20-$45k/y, >$5k/y), education (<HS, HS or tech college, college graduate), abnormal vaginal discharge at birth (yes/no), Group B streptococcus infection at sampling (yes/no) and history of previous preterm birth (yes/no).

## Discussion

In this case-control analysis nested in the CANDLE cohort, we found no difference in urinary microbiota α- or β-diversity between cases and controls. However, OTUs classified to *Prevotella*, *Sutterella*, *L*. *iners*, *Blautia*, *Kocuria*, Lachnospiraceae, and *S*.*marcescens* were enriched among cases delivering prior to 37 weeks gestation. A urinary microbiota clustering partition dominated by *S*. *marcescens* was also associated with PTB. Together, these results provide little support that the composition of the urinary microbiota in this cohort of predominantly non-Hispanic black women as measured by 16S rRNA gene sequencing during the second trimester is associated with PTB. Enrichment among cases in several organisms previously reported to be associated with genitourinary pathology is intriguing, but requires confirmation in future studies.

We observed a diverse microbiota in urine of mothers collected during the second trimester. This is consistent with previous reports demonstrating high diversity in the urine of asymptomatic, culture-negative women.[22–27] The relative abundance of dominant taxa and OTU richness in our sample were similar to those reported by Siddiqui et al.[23] despite differences in the study population, PCR primers, and sequencing technology. Differences in the relative abundance of *Lactobacillus* and *Gardnerella* may, in part, reflect differences in the abilities of primers to amplify *Lactobacillus* and the high number of unclassified Bifidobacteriaceae reads, many expected to be *Gardnerella* organisms, in our study. Our ability to compare these findings to culture or PCR-based studies implicating BV-associated bacteria in the etiology of PTB [16, 54] is limited by the lack of robust species-level resolution inherent to 16S rRNA gene (i.e. amplicon) sequencing. Future efforts utilizing shotgun metagenomic sequencing will provide for further insight into associations for specific bacterial species. An extensive study of the female genital microbiota associated with PTB is currently underway as part of the Integrative Human Microbiome Project (iHMP) and expected to provide significant contributions in this area.[55]

Using an unsupervised clustering approach, we identified four urinary microbiota partitions in a racially and socioeconomically diverse sample of women. Partition 3 was found to be uniquely dominated by *S*. *marcescens* and associated with PTB. *S*. *marcescens* is a non-endospore forming, rod-shaped, gram-negative bacteria and known human opportunistic pathogen causing UTIs, indwelling urinary catheter infections, and important source of hospital acquired infections.[56–58] It is also found in diverse environments, resistant to a wide range of antibiotics, and produces virulence factors associated with cell toxicity, host inflammatory response, and tissue penetration; [57, 59–61] providing a plausible mechanism by which it may influence the risk of PTB. DiGiulio and Callahan et al.[19] reported a high diversity, *Lactobacillus* poor vaginal community type identified using an unsupervised clustering approach to be associated with PTB. Interestingly, both the duration and proportion of time the microbiota were in this community state was associated with PTB; however, it also was the least stable requiring a frequent sampling interval to estimate the portion of time spent in the high diversity state. Conversely, no association with PTB was reported by Romero et al.[11] for similar vaginal community state types examined across pregnancy. Discrepancies across could be due, in part, to differences in the urinary and vaginal microbiota, choice of primers, sequencing technologies, and informatics/statistical approaches; and/or differences in racial/ethnic composition of participants given demonstrated differences in the vaginal microbial by race.[62]

Several individual OTUs and MED nodes, including those mapping to *S*. *marcescens*, were also associated with PTB. *L*. *iners* has been shown to be a dominant member of the vaginal microbiota [62] and was found here to be enriched in preterm samples. *In vitro* studies have suggested *L*. *iners* may induce IL-8 secretion [63] moderating localized proinflammatory activity. *Prevotella* and *Streptococcus* spp. are commonly found in the vagina, potentially uropathogenic, associated with UTIs and BV, [64–67] and were more abundant in samples from mothers delivering preterm. This is in contrast to a recent study in African American women reporting the abundance of *Prevotella* to be inversely associated with risk of PTB.[21] Similarly, the lower vaginal microbiota diversity suggested for women delivering preterm by Nelson et al.[21] was not seen here. It is also noteworthy that OTUs/nodes classified to *F*. *prausnitzii*, *Blautia*, *Ruminococcus*, *and Sutterella* were also associated with PTB. These organisms are found in high abundance in human fecal samples and may suggest pathogenic transmission from the rectum to the genitourinary tract.[68, 69] However, we cannot rule out that their presence does not reflect fecal contamination occurring during collection.

OTUs or MED nodes classified to *Lactobacillus*, *Shuttleworthia*, and *A*. *vaginae* were inversely related to PTB in our sample. *Lactobacillus* was the dominant genus observed in cases and controls; comprising approximately 25% of all sequence reads. *Lactobacillus* dominance is generally considered characteristic of a “healthy” vaginal microbiota with reductions, and relative increases in facultative or anaerobic organisms, associated with dysbiosis.[70] Given that no difference was seen between cases and controls for total *Lactobacillus* reads, this may represent a chance finding and requires confirmation in future studies.

PCR and standard urine culture are limited to identifying only a fraction of true bacterial diversity. NGS platforms now offer inexpensive, high throughput approaches to interrogate unculturable and rare organisms and their role in PTB. To the best of our knowledge, this study represents the first attempt to employ NGS sequencing to examine the urinary microbiota in relation to PTB and provides support that potential uropathogens can be detected in the urine of women delivering preterm. Collection of urine specimens is inexpensive and non-invasive and can be performed over the course of pregnancy facilitating the examination of changes in specific bacteria or community structure over time. In addition, the urine is an environment distinct from that of the vagina, cervix, and rectum and may serve as a valuable biomarker of urogenital microbial communities of potential importance to preterm delivery. Integrated profiling of the genitourinary (e.g. urine, vagina, cervix) and amniotic microbiota across pregnancy will be best suited to address the role of these communities in PTB.

Strengths of the current study include the racial and socioeconomic diversity of participants, access to detailed demographic and clinical information, use of 16S rRNA gene sequencing, and the relativity large number of preterm births. There were also limitations. First, the number of cases and controls when examined across levels of urinary microbiota clustering partitions was small contributing to imprecise estimates for associations with PTB. Second, this analysis was also limited to urine specimens collected during the second trimester. This precluded assessment of the urinary microbiota at other time points, the ability to examine associations for changes over time, and shifts in the microbiota arising from normal biologic processes or environmental factors such as antibiotic use to be captured. The collection of urine at different time points could also be a source of bias should the urinary microbiota change over the course of the second trimester and sampling be performed at different times for cases and controls. However, the vaginal microbiota has been shown stability in community composition and diversity when examined across pregnancy [19] and collection was performed similarly for cases and controls. Third, despite providing materials and instructions, there remains a risk of urine contamination during collection from bacteria in the surrounding environment. Fourth, the high variance seen for several of the urinary microbiota partitions reflects the inherent challenge of attempting to cluster high dimensional microbial communities to a lower dimensional space. Of primary interest in assigning samples to partitions was to assess whether we could identify clusters based on similarities in community composition that may better capture the co-occurrence of abundant taxa (i.e. reduce dimensionality). To the extent that the urinary microbiota is not comprised of distinct taxonomic subgroups, clustering is expected to result in partition heterogeneity, high variance, and reduced ability to detect associations in subsequent inferential tests. Despite these limitations, the results obtained here suggest the potential to identify subgroups of patients at increased risk of PTB. Future studies employing finer taxonomic profiling or shotgun metagenomics are expected to be better suited to identifying more homogenous subgroups of patients. Lastly, despite the advantages 16S rRNA gene sequencing provides over PCR and culture-based approaches, it is limited in the ability to discriminate between similar organisms (i.e. provide species or strain level resolution) and does not provide direct information on the metagenome or microbial gene expression in a given environment.

## Conclusion

Our findings suggest that the composition of the urinary microbiota as measured during the second trimester of pregnancy is not associated with PTB. However, the enrichment of several organisms previously reported to be associated with genitourinary pathology in the urine of mothers delivering preterm warrants further investigation and confirmation. Whole genome sequencing and metatranscriptomic approaches are required to resolve associations for specific organisms at finer taxonomic levels and to assess microbial function in relation to PTB. Should these findings be reproduced they may suggest strategies for the targeting of specific organisms for the prevention of PTB.

## Supporting Information

S1 Table Wilcoxon rank-sum test for differences in α-diversity metrics according to delivery status. (DOCX)

S2 Table Permutational ANOVA of β-diversity distance matrices according to delivery status. (DOCX)

S3 Table Log2-fold differences for UCLUST OTUs according to delivery status. (DOCX)

S4 Table Log2-fold differences for MED nodes according to delivery status. (DOCX)

S5 Table Abundant taxa according to urinary microbiota partition. (DOCX)

## References

[1] Hamilton BE, Hoyert DL, Martin JA, Strobino DM, Guyer B. Annual summary of vital statistics: 2010–2011. Pediatrics. 2013;131(3):548–58. 10.1542/peds.2012-3769 . 23400611 PMC5754931

[2] Blencowe H, Cousens S, Chou D, Oestergaard M, Say L, Moller AB, et al Born too soon: the global epidemiology of 15 million preterm births. Reproductive health. 2013;10 Suppl 1:S2 10.1186/1742-4755-10-S1-S2 24625129 PMC3828585

[3] Liu L, Johnson HL, Cousens S, Perin J, Scott S, Lawn JE, et al Global, regional, and national causes of child mortality: an updated systematic analysis for 2010 with time trends since 2000. Lancet. 2012;379(9832):2151–61. 10.1016/S0140-6736(12)60560-1 . 22579125

[4] Lawn JE, Kerber K, Enweronu-Laryea C, Cousens S. 3.6 million neonatal deaths—what is progressing and what is not? Seminars in perinatology. 2010;34(6):371–86. 10.1053/j.semperi.2010.09.011 . 21094412

[5] Mazor-Dray E, Levy A, Schlaeffer F, Sheiner E. Maternal urinary tract infection: is it independently associated with adverse pregnancy outcome? The journal of maternal-fetal & neonatal medicine: the official journal of the European Association of Perinatal Medicine, the Federation of Asia and Oceania Perinatal Societies, the International Society of Perinatal Obstet. 2009;22(2):124–8. 10.1080/14767050802488246 . 19085630

[6] Schieve LA, Handler A, Hershow R, Persky V, Davis F. Urinary tract infection during pregnancy: its association with maternal morbidity and perinatal outcome. American journal of public health. 1994;84(3):405–10. 8129056 10.2105/ajph.84.3.405 PMC1614832

[7] Averbuch B, Mazor M, Shoham-Vardi I, Chaim W, Vardi H, Horowitz S, et al Intra-uterine infection in women with preterm premature rupture of membranes: maternal and neonatal characteristics. European journal of obstetrics, gynecology, and reproductive biology. 1995;62(1):25–9. . 7493703 10.1016/0301-2115(95)02176-8

[8] Leitich H, Kiss H. Asymptomatic bacterial vaginosis and intermediate flora as risk factors for adverse pregnancy outcome. Best practice & research Clinical obstetrics & gynaecology. 2007;21(3):375–90. 10.1016/j.bpobgyn.2006.12.005 . 17241817

[9] Jacobsson B, Mattsby-Baltzer I, Andersch B, Bokstrom H, Holst RM, Wennerholm UB, et al Microbial invasion and cytokine response in amniotic fluid in a Swedish population of women in preterm labor. Acta obstetricia et gynecologica Scandinavica. 2003;82(2):120–8. . 12648172 10.1034/j.1600-0412.2003.00047.x

[10] Romero R, Espinoza J, Kusanovic JP, Gotsch F, Hassan S, Erez O, et al The preterm parturition syndrome. BJOG: an international journal of obstetrics and gynaecology. 2006;113 Suppl 3:17–42. 10.1111/j.1471-0528.2006.01120.x . 17206962 PMC7062298

[11] Romero R, Hassan SS, Gajer P, Tarca AL, Fadrosh DW, Bieda J, et al The vaginal microbiota of pregnant women who subsequently have spontaneous preterm labor and delivery and those with a normal delivery at term. Microbiome. 2014;2:18 10.1186/2049-2618-2-18 24987521 PMC4066267

[12] Aagaard K, Ma J, Antony KM, Ganu R, Petrosino J, Versalovic J. The placenta harbors a unique microbiome. Science translational medicine. 2014;6(237):237ra65 10.1126/scitranslmed.3008599 . 24848255 PMC4929217

[13] Han YW, Ikegami A, Bissada NF, Herbst M, Redline RW, Ashmead GG. Transmission of an uncultivated Bergeyella strain from the oral cavity to amniotic fluid in a case of preterm birth. Journal of clinical microbiology. 2006;44(4):1475–83. 10.1128/JCM.44.4.1475-1483.2006 16597879 PMC1448680

[14] Fardini Y, Chung P, Dumm R, Joshi N, Han YW. Transmission of diverse oral bacteria to murine placenta: evidence for the oral microbiome as a potential source of intrauterine infection. Infection and immunity. 2010;78(4):1789–96. 10.1128/IAI.01395-09 20123706 PMC2849412

[15] Payne MS, Bayatibojakhi S. Exploring preterm birth as a polymicrobial disease: an overview of the uterine microbiome. Frontiers in immunology. 2014;5:595 10.3389/fimmu.2014.00595 25505898 PMC4245917

[16] Mysorekar IU, Cao B. Microbiome in parturition and preterm birth. Seminars in reproductive medicine. 2014;32(1):50–5. 10.1055/s-0033-1361830 . 24390921

[17] Simcox R, Sin WT, Seed PT, Briley A, Shennan AH. Prophylactic antibiotics for the prevention of preterm birth in women at risk: a meta-analysis. The Australian & New Zealand journal of obstetrics & gynaecology. 2007;47(5):368–77. 10.1111/j.1479-828X.2007.00759.x . 17877593

[18] Hyman RW, Fukushima M, Jiang H, Fung E, Rand L, Johnson B, et al Diversity of the vaginal microbiome correlates with preterm birth. Reproductive sciences. 2014;21(1):32–40. 10.1177/1933719113488838 23715799 PMC3857766

[19] DiGiulio DB, Callahan BJ, McMurdie PJ, Costello EK, Lyell DJ, Robaczewska A, et al Temporal and spatial variation of the human microbiota during pregnancy. Proceedings of the National Academy of Sciences of the United States of America. 2015;112(35):11060–5. 10.1073/pnas.1502875112 26283357 PMC4568272

[20] Kacerovsky M, Vrbacky F, Kutova R, Pliskova L, Andrys C, Musilova I, et al Cervical microbiota in women with preterm prelabor rupture of membranes. PloS one. 2015;10(5):e0126884 10.1371/journal.pone.0126884 25993616 PMC4439143

[21] Nelson DB, Shin H, Wu J, Dominguez-Bello MG. The Gestational Vaginal Microbiome and Spontaneous Preterm Birth among Nulliparous African American Women. American journal of perinatology. 2016 10.1055/s-0036-1581057 . 27057772

[22] Fouts DE, Pieper R, Szpakowski S, Pohl H, Knoblach S, Suh MJ, et al Integrated next-generation sequencing of 16S rDNA and metaproteomics differentiate the healthy urine microbiome from asymptomatic bacteriuria in neuropathic bladder associated with spinal cord injury. Journal of translational medicine. 2012;10:174 10.1186/1479-5876-10-174 22929533 PMC3511201

[23] Siddiqui H, Nederbragt AJ, Lagesen K, Jeansson SL, Jakobsen KS. Assessing diversity of the female urine microbiota by high throughput sequencing of 16S rDNA amplicons. BMC microbiology. 2011;11:244 10.1186/1471-2180-11-244 22047020 PMC3228714

[24] Lewis DA, Brown R, Williams J, White P, Jacobson SK, Marchesi JR, et al The human urinary microbiome; bacterial DNA in voided urine of asymptomatic adults. Frontiers in cellular and infection microbiology. 2013;3:41 10.3389/fcimb.2013.00041 23967406 PMC3744036

[25] Hilt EE, McKinley K, Pearce MM, Rosenfeld AB, Zilliox MJ, Mueller ER, et al Urine is not sterile: use of enhanced urine culture techniques to detect resident bacterial flora in the adult female bladder. Journal of clinical microbiology. 2014;52(3):871–6. 10.1128/JCM.02876-13 24371246 PMC3957746

[26] Wolfe AJ, Toh E, Shibata N, Rong R, Kenton K, Fitzgerald M, et al Evidence of uncultivated bacteria in the adult female bladder. Journal of clinical microbiology. 2012;50(4):1376–83. 10.1128/JCM.05852-11 22278835 PMC3318548

[27] Brubaker L, Wolfe AJ. The new world of the urinary microbiota in women. American journal of obstetrics and gynecology. 2015 10.1016/j.ajog.2015.05.032 . 26003055 PMC4876712

[28] Volgyi E, Carroll KN, Hare ME, Ringwald-Smith K, Piyathilake C, Yoo W, et al Dietary patterns in pregnancy and effects on nutrient intake in the Mid-South: the Conditions Affecting Neurocognitive Development and Learning in Early Childhood (CANDLE) study. Nutrients. 2013;5(5):1511–30. 10.3390/nu5051511 23645026 PMC3708333

[29] Caporaso JG, Lauber CL, Walters WA, Berg-Lyons D, Lozupone CA, Turnbaugh PJ, et al Global patterns of 16S rRNA diversity at a depth of millions of sequences per sample. Proceedings of the National Academy of Sciences of the United States of America. 2011;108 Suppl 1:4516–22. 10.1073/pnas.1000080107 20534432 PMC3063599

[30] Caporaso JG, Kuczynski J, Stombaugh J, Bittinger K, Bushman FD, Costello EK, et al QIIME allows analysis of high-throughput community sequencing data. Nature methods. 2010;7(5):335–6. 10.1038/nmeth.f.303 20383131 PMC3156573

[31] Andrews S. FastQC: a quality control tool for high throughput sequence data. 2010. Available: http://www.bioinformatics.babraham.ac.uk/projects/fastqc.

[32] Pearson WR, Wood T, Zhang Z, Miller W. Comparison of DNA sequences with protein sequences. Genomics. 1997;46(1):24–36. 10.1006/geno.1997.4995 . 9403055

[33] Edgar RC, Haas BJ, Clemente JC, Quince C, Knight R. UCHIME improves sensitivity and speed of chimera detection. Bioinformatics. 2011;27(16):2194–200. 10.1093/bioinformatics/btr381 21700674 PMC3150044

[34] Edgar RC. Search and clustering orders of magnitude faster than BLAST. Bioinformatics. 2010;26(19):2460–1. 10.1093/bioinformatics/btq461 . 20709691

[35] Wang Q, Garrity GM, Tiedje JM, Cole JR. Naive Bayesian classifier for rapid assignment of rRNA sequences into the new bacterial taxonomy. Applied and environmental microbiology. 2007;73(16):5261–7. 10.1128/AEM.00062-07 17586664 PMC1950982

[36] McDonald D, Price MN, Goodrich J, Nawrocki EP, DeSantis TZ, Probst A, et al An improved Greengenes taxonomy with explicit ranks for ecological and evolutionary analyses of bacteria and archaea. The ISME journal. 2012;6(3):610–8. 10.1038/ismej.2011.139 22134646 PMC3280142

[37] Caporaso JG, Bittinger K, Bushman FD, DeSantis TZ, Andersen GL, Knight R. PyNAST: a flexible tool for aligning sequences to a template alignment. Bioinformatics. 2010;26(2):266–7. 10.1093/bioinformatics/btp636 19914921 PMC2804299

[38] Price MN, Dehal PS, Arkin AP. FastTree 2—approximately maximum-likelihood trees for large alignments. PloS one. 2010;5(3):e9490 10.1371/journal.pone.0009490 20224823 PMC2835736

[39] Bokulich NA, Subramanian S, Faith JJ, Gevers D, Gordon JI, Knight R, et al Quality-filtering vastly improves diversity estimates from Illumina amplicon sequencing. Nature methods. 2013;10(1):57–9. 10.1038/nmeth.2276 23202435 PMC3531572

[40] Langille MG, Zaneveld J, Caporaso JG, McDonald D, Knights D, Reyes JA, et al Predictive functional profiling of microbial communities using 16S rRNA marker gene sequences. Nature biotechnology. 2013;31(9):814–21. 10.1038/nbt.2676 23975157 PMC3819121

[41] Shannon CE. The mathematical theory of communication. 1963. MD computing: computers in medical practice. 1997;14(4):306–17. . 9230594

[42] Magurran AE. Measuring biological diversity. Malden, Ma: Blackwell Pub.; 2004 viii, 256 p. p.

[43] Lozupone C, Knight R. UniFrac: a new phylogenetic method for comparing microbial communities. Applied and environmental microbiology. 2005;71(12):8228–35. 10.1128/AEM.71.12.8228-8235.2005 16332807 PMC1317376

[44] Bray JR, Curtis JT. An ordination of upland forest communities of southern Wisconsin. Ecological Monographs. 1957;27:325–49.

[45] Horn H. Measurement of "overlap" in comparative ecological studies. Am Nat. 1966;100:419–24.

[46] Jari Oksanen, F. Guillaume Blanchet, Roeland Kindt, Pierre Legendre, Peter R. Minchin, R. B. O'Hara, et al. vegan: Community Ecology Package. R package version 2.3–0. 2015.

[47] Eren AM, Morrison HG, Lescault PJ, Reveillaud J, Vineis JH, Sogin ML. Minimum entropy decomposition: unsupervised oligotyping for sensitive partitioning of high-throughput marker gene sequences. The ISME journal. 2015;9(4):968–79. 10.1038/ismej.2014.195 . 25325381 PMC4817710

[48] La Rosa PS, Brooks JP, Deych E, Boone EL, Edwards DJ, Wang Q, et al Hypothesis testing and power calculations for taxonomic-based human microbiome data. PloS one. 2012;7(12):e52078 10.1371/journal.pone.0052078 23284876 PMC3527355

[49] Love MI, Huber W, Anders S. Moderated estimation of fold change and dispersion for RNA-seq data with DESeq2. Genome biology. 2014;15(12):550 10.1186/s13059-014-0550-8 25516281 PMC4302049

[50] McMurdie PJ, Holmes S. phyloseq: an R package for reproducible interactive analysis and graphics of microbiome census data. PloS one. 2013;8(4):e61217 10.1371/journal.pone.0061217 23630581 PMC3632530

[51] Holmes I, Harris K, Quince C. Dirichlet multinomial mixtures: generative models for microbial metagenomics. PloS one. 2012;7(2):e30126 10.1371/journal.pone.0030126 22319561 PMC3272020

[52] Schloss PD, Westcott SL, Ryabin T, Hall JR, Hartmann M, Hollister EB, et al Introducing mothur: open-source, platform-independent, community-supported software for describing and comparing microbial communities. Applied and environmental microbiology. 2009;75(23):7537–41. 10.1128/AEM.01541-09 19801464 PMC2786419

[53] Honaker J, King G, Blackwell M. Amelia II: A Program for Missing Data. Journal of Statistical Software. 2011;45(7):1–47.

[54] Brocklehurst P, Gordon A, Heatley E, Milan SJ. Antibiotics for treating bacterial vaginosis in pregnancy. The Cochrane database of systematic reviews. 2013;1:CD000262 10.1002/14651858.CD000262.pub4 . 23440777 PMC11307253

[55] Integrative Human Microbiome Project Research Network Consortium. The Integrative Human Microbiome Project: dynamic analysis of microbiome-host omics profiles during periods of human health and disease. Cell host & microbe. 2014;16(3):276–89. 10.1016/j.chom.2014.08.014 . 25211071 PMC5109542

[56] Hejazi A, Falkiner FR. Serratia marcescens. Journal of medical microbiology. 1997;46(11):903–12. 10.1099/00222615-46-11-903 . 9368530

[57] Mahlen SD. Serratia infections: from military experiments to current practice. Clinical microbiology reviews. 2011;24(4):755–91. 10.1128/CMR.00017-11 21976608 PMC3194826

[58] Kawecki D, Kwiatkowski A, Sawicka-Grzelak A, Durlik M, Paczek L, Chmura A, et al Urinary tract infections in the early posttransplant period after kidney transplantation: etiologic agents and their susceptibility. Transplantation proceedings. 2011;43(8):2991–3. 10.1016/j.transproceed.2011.09.002 . 21996207

[59] Hertle R. The family of Serratia type pore forming toxins. Current protein & peptide science. 2005;6(4):313–25. . 16101433 10.2174/1389203054546370

[60] Shanks RM, Stella NA, Kalivoda EJ, Doe MR, O'Dee DM, Lathrop KL, et al A Serratia marcescens OxyR homolog mediates surface attachment and biofilm formation. Journal of bacteriology. 2007;189(20):7262–72. 10.1128/JB.00859-07 17675374 PMC2168423

[61] Stock I, Grueger T, Wiedemann B. Natural antibiotic susceptibility of strains of Serratia marcescens and the S. liquefaciens complex: S. liquefaciens sensu stricto, S. proteamaculans and S. grimesii. International journal of antimicrobial agents. 2003;22(1):35–47. . 12842326 10.1016/s0924-8579(02)00163-2

[62] Ravel J, Gajer P, Abdo Z, Schneider GM, Koenig SS, McCulle SL, et al Vaginal microbiome of reproductive-age women. Proceedings of the National Academy of Sciences of the United States of America. 2011;108 Suppl 1:4680–7. 10.1073/pnas.1002611107 20534435 PMC3063603

[63] Anahtar MN, Byrne EH, Doherty KE, Bowman BA, Yamamoto HS, Soumillon M, et al Cervicovaginal bacteria are a major modulator of host inflammatory responses in the female genital tract. Immunity. 2015;42(5):965–76. 10.1016/j.immuni.2015.04.019 25992865 PMC4461369

[64] Hay PE. Bacterial Vaginosis as a Mixed Infection In: Brogden KA, G JM, editors. Polymicrobial Diseases. Washington DC: ASM Press; 2002. 21735561

[65] Austin MN, Beigi RH, Meyn LA, Hillier SL. Microbiologic response to treatment of bacterial vaginosis with topical clindamycin or metronidazole. Journal of clinical microbiology. 2005;43(9):4492–7. 10.1128/JCM.43.9.4492-4497.2005 16145097 PMC1234077

[66] Brook I. Urinary tract and genito-urinary suppurative infections due to anaerobic bacteria. International journal of urology: official journal of the Japanese Urological Association. 2004;11(3):133–41. . 15009360 10.1111/j.1442-2042.2003.00756.x

[67] Ulett GC, Webb RI, Ulett KB, Cui X, Benjamin WH, Crowley M, et al Group B Streptococcus (GBS) urinary tract infection involves binding of GBS to bladder uroepithelium and potent but GBS-specific induction of interleukin 1alpha. The Journal of infectious diseases. 2010;201(6):866–70. 10.1086/650696 . 20132033

[68] El Aila NA, Tency I, Claeys G, Verstraelen H, Saerens B, Santiago GL, et al Identification and genotyping of bacteria from paired vaginal and rectal samples from pregnant women indicates similarity between vaginal and rectal microflora. BMC infectious diseases. 2009;9:167 10.1186/1471-2334-9-167 19828036 PMC2770471

[69] Petricevic L, Domig KJ, Nierscher FJ, Krondorfer I, Janitschek C, Kneifel W, et al Characterisation of the oral, vaginal and rectal Lactobacillus flora in healthy pregnant and postmenopausal women. European journal of obstetrics, gynecology, and reproductive biology. 2012;160(1):93–9. 10.1016/j.ejogrb.2011.10.002 . 22088236

[70] Ling Z, Kong J, Liu F, Zhu H, Chen X, Wang Y, et al Molecular analysis of the diversity of vaginal microbiota associated with bacterial vaginosis. BMC genomics. 2010;11:488 10.1186/1471-2164-11-488 20819230 PMC2996984

