# Supplementary material for: Urinary Microbiota Associated with Preterm Birth: Results from the Conditions Affecting Neurocognitive Development and Learning in Early Childhood (CANDLE) Study
Source: PLoS One. 2016 Sep 9;11(9):e0162302. doi: 10.1371/journal.pone.0162302 (PMC5017737; doi:10.1371/journal.pone.0162302)
Supplement: S1 Table — (DOCX) [file pone.0162302.s001.docx]

| Supplemental Table 1. Wilcoxon rank-sum test for differences in α-diversity metrics according to delivery status | | | |  |
| --- | --- | --- | --- | --- |
|  |  |  |  |  |
| Metric | Preterm Median (IQR) | Term Median (IQR) | *P* value |  |
| Observed richness | 275 (84) | 280 (103) | 0.99 |  |
| Shannon index | 4.2 (2.4) | 4.5 (2.5) | 0.94 |  |
| PD Whole Tree (Faith's) | 24.1 (5.0) | 24.2 (6.6) | 0.88 |  |
| Abbreviations: IQR, interquartile range. | | | |  |
| Note: Estimates for UCLUST OTUs (97% similarity). | |  |  |  |
|  | |  |  |  |
