# Supplementary material for: Urinary Microbiota Associated with Preterm Birth: Results from the Conditions Affecting Neurocognitive Development and Learning in Early Childhood (CANDLE) Study
Source: PLoS One. 2016 Sep 9;11(9):e0162302. doi: 10.1371/journal.pone.0162302 (PMC5017737; doi:10.1371/journal.pone.0162302)
Supplement: S2 Table — (DOCX) [file pone.0162302.s002.docx]

| Supplemental Table 2. Permutational ANOVA of β-diversity distance matrices according to delivery status | | | |
| --- | --- | --- | --- |
|  |  |  |  |
|  | F model | R^2^ | *P* value* |
| **UCLUST OTUs** |  |  |  |
| Weighted UniFrac | 0.506 | 0.005 | 0.78 |
| Unweighted UniFrac | 0.728 | 0.008 | 0.85 |
| Bray-Curtis | 0.770 | 0.008 | 0.68 |
| Jaccard | 0.824 | 0.009 | 0.48 |
| Morisita-Horn | 0.916 | 0.010 | 0.47 |
| **MED Nodes** |  |  |  |
| Bray-Curtis | 0.805 | 0.008 | 0.69 |
| Jaccard | 0.873 | 0.009 | 0.68 |
| Morisita-Horn | 0.967 | 0.010 | 0.47 |
| **Imputed Metagenome** |  |  |  |
| Bray-Curtis | 0.430 | 0.005 | 0.82 |
| Jaccard | 0.532 | 0.006 | 0.86 |
| Morisita-Horn | 0.640 | 0.007 | 0.51 |
| Abbreviations: ANOVA, analysis of variance; OTU, operational taxonomic unit; MED, Minimum Entropy Decomposition. | | | |
| Notes: Permutational ANOVA as implemented by the ADONIS function in the R package vegan. | | | |
| **P* value obtained from 1000 permutations of the raw data. | |  |  |
