# Supplementary material for: Urinary Microbiota Associated with Preterm Birth: Results from the Conditions Affecting Neurocognitive Development and Learning in Early Childhood (CANDLE) Study
Source: PLoS One. 2016 Sep 9;11(9):e0162302. doi: 10.1371/journal.pone.0162302 (PMC5017737; doi:10.1371/journal.pone.0162302)
Supplement: S3 Table — (DOCX) [file pone.0162302.s003.docx]

| Supplemental Table 3. Log2-fold differences for UCLUST OTUs according to delivery status. | | | | | | |  |  |  |  |
| --- | --- | --- | --- | --- | --- | --- | --- | --- | --- | --- |
|  |  |  |  |  |  |  |  |  |  |  |
| **OTUID** | **baseMean** | **log2FoldChange** | **lfcSE** | ***P* value*** | **Phylum** | **Class** | **Order** | **Family** | **Genus** | **Species** |
| denovo18959 | 185.839 | 2.416 | 0.730 | 0.023 | Bacteroidetes | Bacteroidia | Bacteroidales | Prevotellaceae | Prevotella | NA |
| denovo72400 | 333.842 | 2.161 | 0.698 | 0.031 | Bacteroidetes | Bacteroidia | Bacteroidales | Prevotellaceae | Prevotella | NA |
| denovo68516 | 19.232 | 1.744 | 0.585 | 0.040 | Proteobacteria | Betaproteobacteria | Burkholderiales | Alcaligenaceae | Sutterella | NA |
| denovo30044 | 48.212 | 1.520 | 0.463 | 0.023 | Firmicutes | Clostridia | Clostridiales | Lachnospiraceae | NA | NA |
| denovo15762 | 111.666 | 1.437 | 0.443 | 0.023 | Firmicutes | Clostridia | Clostridiales | Ruminococcaceae | Faecalibacterium | prausnitzii |
| denovo20462 | 96.341 | 1.360 | 0.422 | 0.023 | Firmicutes | Bacilli | Lactobacillales | Streptococcaceae | Streptococcus | NA |
| denovo79963 | 38.841 | 1.344 | 0.384 | 0.019 | Actinobacteria | Actinobacteria | Actinomycetales | Micrococcaceae | Kocuria | NA |
| denovo31907 | 316.805 | -1.119 | 0.340 | 0.023 | Actinobacteria | Actinobacteria | Bifidobacteriales | Bifidobacteriaceae | Bifidobacterium | NA |
| denovo27688 | 26.456 | -1.373 | 0.453 | 0.036 | Firmicutes | Clostridia | Clostridiales | Veillonellaceae | Megasphaera | NA |
| denovo23506 | 119.367 | -1.557 | 0.366 | 0.002 | Firmicutes | Bacilli | Lactobacillales | Streptococcaceae | Streptococcus | NA |
| denovo26929 | 408.819 | -1.648 | 0.494 | 0.023 | Firmicutes | Clostridia | Clostridiales | [Tissierellaceae] | Peptoniphilus | NA |
| denovo27138 | 151.212 | -2.443 | 0.698 | 0.019 | Actinobacteria | Coriobacteriia | Coriobacteriales | Coriobacteriaceae | Atopobium | vaginae |
| denovo14058 | 18.792 | -2.623 | 0.706 | 0.014 | Firmicutes | Bacilli | Lactobacillales | Streptococcaceae | Streptococcus | agalactiae |
| denovo71704 | 19.487 | -3.521 | 1.096 | 0.023 | Firmicutes | Clostridia | Clostridiales | Veillonellaceae | Veillonella | NA |
| denovo23765 | 4162.015 | -4.245 | 0.658 | <0.001 | Firmicutes | Clostridia | Clostridiales | Lachnospiraceae | Shuttleworthia | NA |
| Abbreviations: lfcSE, log2-fold change standard error; OTU, operational taxonomic unit. | | | | | | |  |  |  |  |
| Notes: OTU clustering at 97% similarity. Log2-fold change for preterm vs. term delivery. Estimates obtained from negative-binomial regression as implemented in the R package DESeq2. | | | | | | | | | | |
| *Benjamini and Hochberg false discovery rate corrected p-value. | | | | |  |  |  |  |  |  |
