# Supplementary material for: Urinary Microbiota Associated with Preterm Birth: Results from the Conditions Affecting Neurocognitive Development and Learning in Early Childhood (CANDLE) Study
Source: PLoS One. 2016 Sep 9;11(9):e0162302. doi: 10.1371/journal.pone.0162302 (PMC5017737; doi:10.1371/journal.pone.0162302)
Supplement: S4 Table — (DOCX) [file pone.0162302.s004.docx]

| Supplemental Table 4. Log2-fold differences for MED nodes according to delivery status. | | | | | | |  |  |  |  |
| --- | --- | --- | --- | --- | --- | --- | --- | --- | --- | --- |
|  |  |  |  |  |  |  |  |  |  |  |
| **nodeID** | **baseMean** | **log2FoldChange** | **lfcSE** | ***P* value*** | **Phylum** | **Class** | **Order** | **Family** | **Genus** | **Species** |
| node14809 | 14.043 | 2.809 | 0.805 | 0.025 | Firmicutes | Bacilli | Lactobacillales | Lactobacillaceae | Lactobacillus | iners |
| node8746 | 41.664 | 2.751 | 0.741 | 0.014 | Proteobacteria | Betaproteobacteria | Burkholderiales | Alcaligenaceae | Parasutterella | excrementihominis |
| node9573 | 27.949 | 2.441 | 0.626 | 0.010 | Firmicutes | Clostridia | Clostridiales | Lachnospiraceae | Blautia | obeum |
| node9484 | 41.171 | 2.221 | 0.676 | 0.035 | Firmicutes | Clostridia | Clostridiales | Lachnospiraceae | Ruminococcus |  |
| node3743 | 46.959 | 2.063 | 0.616 | 0.035 | Bacteroidetes | Bacteroidia | Bacteroidales | Prevotellaceae | Prevotella | copri |
| node10645 | 13.139 | 1.958 | 0.538 | 0.017 | Proteobacteria | Gammaproteobacteria | Pseudomonadales | Pseudomonadaceae Pseudomonas | |  |
| node3853 | 190.324 | 1.414 | 0.453 | 0.042 | Firmicutes | Clostridia | Clostridiales | Lachnospiraceae | Blautia |  |
| node4417 | 9.237 | 0.904 | 0.307 | 0.050 | Proteobacteria | Gammaproteobacteria | Enterobacteriales | Enterobacteriaceae | Serratia | marcescens |
| node10887 | 139.017 | -0.949 | 0.308 | 0.043 | Proteobacteria | Alphaproteobacteria | Rhodobacterales | Rhodobacteraceae Pannonibacter | | phragmitetus |
| node6535 | 60.847 | -1.234 | 0.397 | 0.042 | Firmicutes | Bacilli | Lactobacillales | Streptococcaceae | Streptococcus | mitis |
| node59 | 90.903 | -1.272 | 0.410 | 0.042 | Firmicutes | Bacilli | Lactobacillales | Lactobacillaceae | Lactobacillus |  |
| node5427 | 314.023 | -1.277 | 0.404 | 0.042 | Thermi | Deinococci | Thermales | Thermaceae | Thermus |  |
| node2606 | 49.646 | -1.541 | 0.448 | 0.027 | Proteobacteria | Gammaproteobacteria | Alteromonadales | Shewanellaceae | Symbiobacterium | thermophilum |
| node15178 | 1674.994 | -1.800 | 0.578 | 0.042 | Firmicutes | Bacilli | Lactobacillales | Lactobacillaceae | Lactobacillus |  |
| node5266 | 823.203 | -2.172 | 0.695 | 0.042 | Actinobacteria | Coriobacteriia | Coriobacteriales | Coriobacteriaceae | Atopobium | vaginae |
| node12688 | 1935.823 | -2.282 | 0.696 | 0.035 | Actinobacteria | Coriobacteriia | Coriobacteriales | Coriobacteriaceae | Atopobium | vaginae |
| node13390 | 11.401 | -2.524 | 0.838 | 0.046 | Actinobacteria | Actinobacteria | Bifidobacteriales | Bifidobacteriaceae | Gardnerella |  |
| node14369 | 14.229 | -2.612 | 0.863 | 0.046 | Actinobacteria | Actinobacteria | Bifidobacteriales | Bifidobacteriaceae | Gardnerella |  |
| node7423 | 29.332 | -2.680 | 0.812 | 0.035 | Actinobacteria | Actinobacteria | Bifidobacteriales | Bifidobacteriaceae | Gardnerella |  |
| node7432 | 24.105 | -2.740 | 0.845 | 0.038 | Actinobacteria | Actinobacteria | Bifidobacteriales | Bifidobacteriaceae | Gardnerella |  |
| node12230 | 11.456 | -2.744 | 0.885 | 0.042 | Actinobacteria | Actinobacteria | Actinomycetales | Corynebacteriaceae | Corynebacterium |  |
| node13383 | 23.372 | -2.794 | 0.914 | 0.044 | Actinobacteria | Actinobacteria | Bifidobacteriales | Bifidobacteriaceae | Gardnerella |  |
| node14086 | 15.581 | -2.803 | 0.899 | 0.042 | Actinobacteria | Actinobacteria | Bifidobacteriales | Bifidobacteriaceae | Gardnerella |  |
| node12779 | 29.606 | -3.005 | 0.978 | 0.043 | Firmicutes | Clostridia | Clostridiales | Lachnospiraceae | Shuttleworthia |  |
| node14088 | 19.248 | -3.147 | 0.943 | 0.035 | Actinobacteria | Actinobacteria | Bifidobacteriales | Bifidobacteriaceae | Gardnerella |  |
| node7425 | 18.938 | -3.219 | 0.902 | 0.020 | Actinobacteria | Actinobacteria | Bifidobacteriales | Bifidobacteriaceae | Gardnerella |  |
| node11215 | 135.685 | -3.455 | 0.819 | 0.004 | Firmicutes | Bacilli | Lactobacillales | Aerococcaceae | Aerococcus | urinae |
| node13667 | 39.186 | -3.549 | 1.108 | 0.042 | Firmicutes | Clostridia | Clostridiales | Lachnospiraceae | Shuttleworthia |  |
| node997 | 15.819 | -3.781 | 0.824 | 0.001 | Firmicutes | Bacilli | Lactobacillales | Streptococcaceae | Streptococcus | agalactiae |
| node6157 | 106.683 | -3.836 | 0.889 | 0.003 | Firmicutes | Clostridia | Clostridiales | Lachnospiraceae | Shuttleworthia |  |
| node12785 | 47.935 | -3.931 | 1.031 | 0.010 | Firmicutes | Clostridia | Clostridiales | Lachnospiraceae | Shuttleworthia |  |
| node14303 | 3673.154 | -3.988 | 0.721 | <0.001 | Firmicutes | Clostridia | Clostridiales | Lachnospiraceae | Shuttleworthia |  |
| node13670 | 36.283 | -4.023 | 1.045 | 0.010 | Firmicutes | Clostridia | Clostridiales | Lachnospiraceae | Shuttleworthia |  |
| node7572 | 39.802 | -4.131 | 1.369 | 0.046 | Actinobacteria | Actinobacteria | Bifidobacteriales | Bifidobacteriaceae | Gardnerella |  |
| node13668 | 28.998 | -4.320 | 1.116 | 0.010 | Firmicutes | Clostridia | Clostridiales | Lachnospiraceae | Shuttleworthia |  |
| Abbreviations: lfcSE, log2-fold change standard error; MED, Minimum Entropy Decomposition. | | | | | | |  |  |  |  |
| Notes: Log2-fold change for preterm vs. term delivery. Estimates obtained from negative-binomial regression as implemented in the R package DESeq2. | | | | | | | | | |  |
| *Benjamini and Hochberg false discovery rate corrected p-value. | | | | | |  |  |  |  |  |
