# Supplementary material for: Urinary Microbiota Associated with Preterm Birth: Results from the Conditions Affecting Neurocognitive Development and Learning in Early Childhood (CANDLE) Study
Source: PLoS One. 2016 Sep 9;11(9):e0162302. doi: 10.1371/journal.pone.0162302 (PMC5017737; doi:10.1371/journal.pone.0162302)
Supplement: S5 Table — (DOCX) [file pone.0162302.s005.docx]

| Supplemental Table 5. Abundant taxa according to urinary microbiota partition | | |  |  |
| --- | --- | --- | --- | --- |
|  |  |  |  |  |
| Taxa | Partition 1 | Partition 2 | Partition 3 | Partition 4 |
| Bifidobacteriaceae ; unclassified | 22.0 | 9.6 | 5.7 | 4.9 |
| Lactobacillus | 7.5 | 29.3 | 10.4 | 72.6 |
| Prevotella | 5.9 | 1.3 | 1.1 | 0.5 |
| Shuttleworthia | 5.8 | 0.3 | 0.3 | 0.1 |
| Atopobium | 4.2 | 1.0 | 0.3 | 0.3 |
| Streptococcus | 4.0 | 0.8 | 1.1 | 0.2 |
| Serratia | 3.3 | 7.5 | 25.6 | 4.8 |
| Megasphaera | 2.9 | 0.3 | 0.2 | 0.2 |
| Bifidobacterium | 2.4 | 0.7 | 0.5 | 0.2 |
| Caulobacteraceae; unclassified | 2.4 | 6.3 | 7.4 | 1.5 |
| Paenibacillus | 2.0 | 0.2 | 0.6 | 0.1 |
| Enterococcus | 1.8 | 0.3 | 0.2 | 0.1 |
| Sneathia | 1.7 | 0.3 | 0.1 | 0.0 |
| Oxalobacteraceae; unclassified | 1.6 | 4.7 | 3.8 | 0.8 |
| Staphylococcus | 1.6 | 1.3 | 1.3 | 0.5 |
| Thermus | 1.4 | 1.3 | 1.3 | 0.2 |
| Acinetobacter | 1.3 | 1.4 | 2.3 | 0.5 |
| Gemella | 1.2 | 0.4 | 0.3 | 0.0 |
| Corynebacterium | 1.1 | 1.4 | 1.1 | 1.5 |
| Bacteroides | 1.1 | 0.8 | 1.1 | 0.4 |
| Allobaculum | 0.9 | 0.9 | 1.3 | 0.3 |
| Gordonia | 0.8 | 1.6 | 3.9 | 0.8 |
| Aerococcus | 0.8 | 0.3 | 0.1 | 0.2 |
| Pseudomonas | 0.7 | 0.6 | 1.1 | 0.3 |
| Notes: Values represent % of total for each partition. Values shown for 25 most abundant genus-level taxa. | | | |  |
